# *WDR4* gene polymorphisms and Wilms tumor susceptibility in Chinese children: A five-center case-control study

**DOI:** 10.7150/jca.83747

**Published:** 2023-05-08

**Authors:** Linqing Deng, Rui-Xi Hua, Changmi Deng, Jinhong Zhu, Zhengtao Zhang, Jiwen Cheng, Jiao Zhang, Haixia Zhou, Suhong Li, Jichen Ruan, Guochang Liu, Jing He, Wen Fu

**Affiliations:** 1 Department of Pediatric Surgery, Guangzhou Institute of Pediatrics, Guangdong Provincial Key Laboratory of Research in Structural Birth Defect Disease, Guangzhou Women and Children's Medical Center, Guangzhou Medical University, Guangdong Provincial Clinical Research Center for Child Health, Guangzhou 510623, Guangdong, China.; 2 Department of Clinical Laboratory, Biobank, Harbin Medical University Cancer Hospital, Harbin 150040, Heilongjiang, China.; 3 Department of Pediatric Surgery, The Second Affiliated Hospital of Xi'an Jiaotong University, Xi'an 710004, Shaanxi, China.; 4 Department of Pediatric Surgery, The First Affiliated Hospital of Zhengzhou University, Zhengzhou 450052, Henan, China.; 5 Department of Hematology, The Key Laboratory of Pediatric Hematology and Oncology Diseases of Wenzhou, The Second Affiliated Hospital and Yuying Children's Hospital of Wenzhou Medical University, Wenzhou 325027, Zhejiang, China.; 6 Department of Pathology, Children Hospital and Women Health Center of Shanxi, Taiyuan 030013, Shannxi, China.

**Keywords:** Wilms tumor, susceptibility, *WDR4*, polymorphism, m7G modification

## Abstract

Wilms tumor is the most common embryonal renal malignancy in children. WDR4 is an indispensable noncatalytic subunit of the RNA N7-methylguanosine (m7G) methyltransferase complex and plays an essential role in tumorigenesis. However, the relationship between polymorphisms in the *WDR4* gene and susceptibility to Wilms tumor remains to be fully investigated. We performed a large case-control study involving 414 patients and 1199 cancer-free controls to investigate whether single nucleotide polymorphisms (SNPs) in the *WDR4* gene are associated with Wilms tumor susceptibility. *WDR4* gene polymorphisms (rs2156315 C > T, rs2156316 C > G, rs6586250 C > T, rs15736 G > A, and rs2248490 C > G) were genotyped using the TaqMan assay. In addition, unconditioned logistic regression analysis was performed, odds ratios (ORs) and 95% confidence intervals (CIs) were used to assess the association between *WDR4* gene SNPs and Wilms tumor susceptibility as well as the strength of the associations. We found that only the rs6586250 C>T polymorphism was significantly associated with an increased risk of Wilms tumor (adjusted OR=2.99, 95% CI = 1.28-6.97, *P* = 0.011 for the rs6586250 TT genotype; adjusted OR=3.08, 95% CI = 1.33-7.17, *P* = 0.009 for the rs6586250 CC/CT genotype). Furthermore, the stratification analysis revealed that patients with the rs6586250 TT genotype and carriers with 1-5 risk genotypes exhibited statistically significant associations with increased Wilms tumor risk in specific subgroups. However, the rs2156315 CT/TT genotype was identified as having a protective effect against Wilms tumor in the age >18 months subgroup compared with the rs2156315 CC genotype. In brief, our study demonstrated that the rs6586250 C > T polymorphism of the *WDR4* gene was significantly associated with Wilms tumor. This finding may contribute to the understanding of the genetic mechanism of Wilms tumor.

## Introduction

Wilms tumor is the most frequently occurring embryonal renal malignancy in children, representing approximately 7% of childhood tumors 1. It often develops in children before the age of 15 years, and the incidence of Wilms tumor varies greatly by geography and ethnicity. The highest incidence rate of Wilms tumor is in the Black population in the United States, with over 10 cases per million children, while the East Asian population has the lowest incidence rate of 3 to 4 cases per million children 2, 3. In China, the annual prevalence of Wilms tumor is approximately 3.3 cases per million children 4. With advances in modern multimodality therapy, the overall survival rate for patients with Wilms tumor has reached more than 90%, except for patients with unfavorable histology, advanced disease, bilateral disease, and recurrent Wilms tumor 5. The outcome is not positive for children with relapsed disease because of a lower long-term survival rate of less than 30% 6. In addition, it has been reported that nearly 25% of Wilms tumor survivors suffer from chronic health problems owing to the side effects of cancer treatment, such as kidney failure, cardiac disease, and secondary malignancies 7, 8.

The interaction of environmental and genetic factors is a crucial cause of tumorigenesis. In previous publications, several perinatal and environmental factors were identified as risk factors for Wilms tumor, including exposure to pesticides during pregnancy, preterm birth, high birthweight and maternal hypertension 9. Rapidly developing biotechnology has led to the identification of an increasing number of Wilms tumor susceptibility genes, including *WT1*, *P53*, *TRIM28*, *BARD1*, *XPD*, *hOGG1*, and *FEN1* 10-15. However, thus far, the well-established genetic alterations can only explain a small part of the tumorigenesis of Wilms tumor. It is imperative to validate additional genetic variants to better understand the pathogenesis of Wilms tumor and develop new drug-targeted therapies.

Misregulation in RNA modification has been found to be closely related to many human diseases, including genetic defects, neurological diseases, and cancers 16. Currently, over 160 kinds of RNA modifications have been discovered, and tRNAs are the most heavily modified 17, 18. The *METTL1/WDR4* complex is an extremely critical tRNA N7-methylguanosine (m7G) methyltransferase that catalyzes m7G modifications not only in the cap of mRNAs but also in internal mRNAs and tRNAs, helping to stabilize the structure of tRNAs and enhance the efficiency of mRNA translation 19, 20. Moreover, in the G-rich regions of miRNAs, m7G appeared to form an unstable structure of G-quadruplexes, facilitating the process of pre-miRNA and miRNA maturation 21. Mammalian m7G modifications display additional complexity and critical physiological functions. *METTL1* and *WDR4* defects have been reported to cause impaired stem cell self-renewal ability and neural differentiation in mouse embryonic stem cells 22, 23. Moreover, *WDR4* was demonstrated to be a potential oncogene. Zeng et al. observed that* WDR4* was aberrantly overexpressed in different tumors, and the expression level of *WDR4* was closely related to poor prognosis in a human pan-cancer analysis of 33 different types of cancers 24. In addition, mutations in the *WDR4* gene cause not only microcephalic primordial dwarfism accompanied by different levels of severe growth retardation and microcephaly 25 but also Galloway-Mowat syndrome, characterized by neurodevelopmental defects and renal-glomerular disease 26. *METTL1* or *WDR4* expression dysregulation has been detected in many tumors, including bladder cancer, head and neck squamous cell carcinoma, hepatocellular carcinoma, and lung cancer 27-30. To date, no research has investigated the association between functional single nucleotide polymorphisms (SNPs) in the *WDR4* gene and Wilms tumor susceptibility.

## Materials and Methods

### Study population

This case-control study enrolled 414 patients with Wilms tumor and 1199 healthy controls as described previously (**Table S1**) 31-33. All Wilms tumor cases were confirmed by histopathology. Age-, gender- and ethnicity-matched healthy controls were enrolled from the same hospitals as the Wilms tumor patients during the same period and had no family history of Wilms tumor. All procedures in this study adhered to the Declaration of Helsinki. We obtained approval from the Institutional Review Board of Guangzhou Women and Children's Medical Center (No. 202016601), as well as written informed consent from the subjects' parents or guardians.

### Genotyping

According to the standard criteria described in previous studies, we selected five potentially functional single nucleotide polymorphisms in the *WDR4* gene (rs2156315 C > T, rs2156316 C > G, rs6586250 C > T, rs15736 G>A, and rs2248490 C > G) from the NCBI dbSNP database (http://www.ncbi.nlm.nih.gov/projects/SNP) and SNPinfo (http://snpinfo.niehs.nih.gov/snpfunc.htm) 32, 34. Briefly, we selected the underlying SNPs in the 5' and 3' untranslated regions, 5' near regions, 3' near regions and exons of the *WDR4* gene. Moreover, the selected SNPs had a minor allele frequency (MAF) ≥5% among Chinese Han subjects and low linkage disequilibrium (LD) (R^2^<0.8). Additionally, SNPs that affect the activity of transcription factor binding site (TFBS) activity or miRNA binding sites were included. By using SNPinfo Web Server (https://snpinfo.niehs.nih.gov/snpinfo/snpfunc.html), we discovered that rs15736 G > A and rs2248490 C > G have the potential to affect the exonic splicing enhancer (ESE) or exonic splicing silencer (ESS), whereas rs2156315 C > T and rs2156316 C > G are likely to affect miRNA binding sites. Finally, rs6586250 C > T, rs15736 G > A and rs2248490 C > G may influence nonsynonymous coding SNPs (nsSNPs) (**Table S2**). DNA was extracted from the peripheral blood samples of participants by the Genomic DNA kit (Tian Gen Biotech Co. Ltd., Beijing, China), and genotyping of *WDR4* gene polymorphisms was carried out by the TaqMan assay. Generally, to ensure the study's accuracy, 10% of all samples were randomly selected for regenotyping by a double-blind method, and the results of regenotyping were consistent with those of the original genotyping.

### Statistical analysis

The consistency between the selected SNP genotype and Hardy-Weinberg Equilibrium (HWE) in controls was determined by a χ^2^ goodness-of-fit test. In addition, a two-sided χ^2^ test was applied to analyze the differences in the distribution of demographic characteristics and allele frequency between cases and cancer-free controls. We also calculated the adjusted odds ratio (OR), 95% confidence interval (CI), and a two-sided *P* value to assess the association between each genotype and the risk of Wilms tumor by multivariate logistic regression analysis. Additionally, expression quantitative trait locus (eQTL) analysis was conducted to evaluate the correlations between significant SNPs and the expression levels of their genes or nearby genes using released data from Genotype-Tissue Expression (GTEx) (https://gtexportal.org). The statistically significant threshold was defined as *P*<0.05. All statistical analyses were implemented with SAS 9.1 software (SAS Institute, Cary, NC, USA).

## Results

### Association between *WDR4* gene polymorphisms and Wilms tumor susceptibility

In our study, 403 cases and 1198 healthy controls were successfully genotyped for the five SNPs out of a cohort of 414 cases and 1199 cancer-free controls. The correlation of *WDR4* gene polymorphisms with Wilms tumor susceptibility is portrayed in **Table 1**. The frequency distribution of all the analyzed SNP genotypes obeyed the HWE in controls. Notably, only the rs6586250 C > T genotype was significantly associated with an increased risk of Wilms tumor (adjusted OR = 2.99, 95% CI = 1.28 - 6.97, *P* = 0.011 for the rs6586250 TT genotype; adjusted OR = 3.08, 95% CI = 1.33-7.17, *P* = 0.009 for the rs6586250 CC/CT genotype). According to the ORs, we subsequently defined rs2156315 TT, rs2156316 CC, rs6586250 TT, rs15736 AA, and rs2248490 CC as risk genotypes. However, the 1-5 combined risk genotypes had no statistically significant effects on Wilms tumor susceptibility compared to non-risk genotypes.

### Stratification analysis

We further employed a stratification analysis divided by age, sex, and clinical stage to explore whether *WDR4* gene polymorphisms were associated with Wilms tumor risk in a specific subgroup, and the corresponding results are shown in **Table 2**. We found a statistically significant association between the rs6586250 TT genotype and increased risk of Wilms tumor in the specific subgroups of children aged over 18 months (adjusted OR = 3.89, 95% CI = 1.33 - 11.37, *P* = 0.013), females (adjusted OR = 7.13, 95% CI = 1.37 - 37.06, *P* = 0.020), and patients with clinical stage I+II (adjusted OR=3.11, 95% CI = 1.19 - 8.15, *P* = 0.021) and clinical stage III+IV (adjusted OR = 3.28, 95% CI = 1.02 - 10.52, *P* = 0.046). Similarly, in children older than 18 months, carriers with 1-5 risk genotypes were more likely to develop Wilms tumor (adjusted OR = 1.45, 95% CI = 1.09-1.92, *P* = 0.011) than carriers without any risk genotype. However, the rs2156315 CT/TT genotype was identified as having a protective effect against Wilms tumor in the specific subgroup of children older than 18 months when compared with the rs2156315 CC genotype (adjusted OR = 0.73, 95% CI = 0.55 - 0.98, *P* = 0.036). This could be due to different SNP genotypes having different effects on the same tumor, or it could be due to chance because of the limited number of subjects. Expanded sample sizes and replicate experiments are strongly recommended.

### Effect of rs6586250 C>T on the expression of the *WDR4* gene and surrounding genes

**Figure 1:**
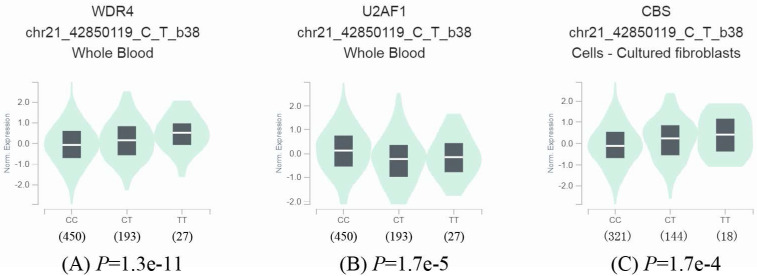
Effect of *WDR4* gene rs6586250 C>T on the expression of the *WDR4* gene and surrounding genes. (A) The expression of the *WDR4* gene in whole blood; (B) The expression of the *U2AF1* gene in whole blood; (C) The expression of the *CBS* gene in cultured fibroblasts cells.

To further identify the effect of rs6586250 C > T on mRNA expression, we took full advantage of the released data from GTEx to perform an eQTL analysis of rs6586250 C > T. As shown in **Figure 1**, in whole blood, compared with the rs6586250 CC genotype, the rs6586250 CT/TT genotype was significantly associated with higher *WDR4* mRNA expression (*P*=1.3e^-11^), whereas it was correlated with lower mRNA expression of the nearby gene *U2AF1* (*P* = 1.7e^-5^). In addition, the rs6586250 CT/TT genotype showed enhanced mRNA expression of the nearby gene *CBS* in cultured fibroblast cells compared to the rs6586250 CC genotype (*P* = 1.7 e^-4^).

## Discussion

RNA modification has been proven to be dynamic and reversible and is involved in many important biological processes, such as cell differentiation, gene expression and protein synthesis 35, 36. Thus, dysregulation of RNA modification may contribute to disease development by disrupting normal biological processes. *WDR4*, a member of the repeat protein family and the yeast ortholog of *Trm82p*, is located at human chromosome 21q22.3 37. WDR4 binds to METTL1 to form a heterodimer complex of m7G methyltransferase, which catalyzes the installation of m7G modification in different types of RNA molecules. m7G modification affects the metabolism and functions of RNA and ultimately controls gene expression and critical biological processes 38. *METTL1* plays the larger part in catalyzing the installation of m7G in target RNA, while *WDR4* helps stabilize the active conformation and maintain the normal protein level of *METTL1*. The integrity of the METTL1/WDR4 complex is considered indispensable for the function of m7G modification 39. The METTL1/WDR4 complex catalyzes the installation of m7G modification in a set of tRNAs and generates the so-called *Arg-TCT* tRNAs, which decode the corresponding homologous mRNA codons and thus selectively promote the translation of many cell cycle-associated and carcinogenic genes. This mechanism greatly contributes to oncogenic transformation 40.

Further studies revealed that METTL1/WDR4-mediated m7G modification played a double-edged role in the pathogenesis and progression of different tumors 41. The main reason for this significant difference largely depended on whether it was mounted on the G46 site of tRNAs or the G-quadruplex structure of miRNAs 41. According to previous research, the expression of *WDR4* transcripts was obviously upregulated in hepatocellular carcinoma (HCC), along with increasing levels of m7G methylation. Overexpression of *WDR4* promoted the development and metastasis of HCC by upregulating the cell cycle-associated factor *CCNB1* and epithelial-mesenchymal transition (EMT) via the MYC/WDR4/CCNB1 signaling pathway. WDR4 plays an oncogenic role in HCC by positively regulating the expression of CCNB1 to induce the G2/M cell cycle transition and inhibit apoptosis, which promotes the proliferation ability of cancer cells 29. In contrast, METTL1/WDR4-mediated m7G-modified microRNAs suppressed the progression of lung cancer. The complex directly methylated a series of tumor-suppressive miRNAs, particularly the *let-7e* family, and promoted the maturation of *let-7e* miRNAs; these miRNAs in return downregulated specific oncogenes such as high-mobility group AT-hook 2 (*HMGA2*), thereby suppressing the migration ability of lung cancer cells 21.

With tremendous advances in high-throughput sequencing technology, genome-wide association studies (GWASs) have successfully identified many predisposition loci that contribute to multiple complex human diseases, including cancers 42. The underlying mechanistic elucidation of SNPs for cancer predisposition is that most nucleotide substitutions occur in noncoding regulatory regions, playing a crucial role in modulating gene expression by affecting RNA splicing and DNA methylation 42-44. For instance, Wang et al. 45 discovered that the rs465663 polymorphism in the *WDR4* gene, located in the intronic region, was related to asthenozoospermia (a clinical manifestation of male infertility) by regulating *WDR4* gene expression and the DNA fragmentation level caused by oxidative stress. He et al. 34 conducted a case-control study of 313 hepatoblastoma patients and 1446 controls to explore whether patients with the *WDR4* gene polymorphism were predisposed to hepatoblastoma in Chinese Han populations. They found that female carriers with 2-5 risk genotypes were more vulnerable to hepatoblastoma, demonstrating that the *WDR4* gene polymorphism may be a risk factor for hepatoblastoma. However, no studies involving the relationship between genetic variants in the *WDR4* gene and Wilms tumor risk have been reported thus far. Herein, our results suggested that *WDR4* gene SNPs predispose patients to Wilms tumor. This finding may make a genetic contribution to the molecular mechanism of Wilms tumor.

In the current study, we demonstrated that the selected rs6586250 C > T polymorphism of the *WDR4* gene was significantly associated with an increased risk of Wilms tumor. Further stratification analysis identified that the rs6586250 TT genotype was associated with a statistically significant increased risk of Wilms tumor in certain subgroups: children older than 18 months, females, and patients in clinical stage I+II and clinical stage III+IV. Similarly, carriers with 1-5 risk genotypes among children aged over 18 months were more likely to develop Wilms tumors compared to those without any risk genotypes. In conclusion, these data suggested that functionally selected SNPs in the *WDR4* gene have a significant impact on increased risk for Wilms tumor. These findings have enriched our knowledge about the etiology of Wilms tumor and facilitated the identification of new biomarker molecules for early diagnosis and good prognosis. Moreover, we tried to further explore the possible mechanism of how significant SNPs of *WDR4* conferred susceptibility to Wilms tumor. According to the eQTL analysis of *WDR4* rs6586250 C>T, we determined that the rs6586250 T allele was significantly associated with higher WDR4 mRNA expression in whole blood. Previous studies also showed an overexpression of WDR4 in head and neck squamous cell carcinoma 28, hepatocellular carcinoma 29, intrahepatic cholangiocarcinoma 46, and so on, indicating that WDR4 serves as an oncogene for several cancers. To date, although there have been no studies on the direct effect of WDR4 on the risk of Wilms tumor, our results suggested that the *WDR4* rs6586250 T genotype may increase the susceptibility to Wilms tumor by increasing *WDR4* gene expression. Additionally, the eQTL analysis also identified that the *WDR4* rs6586250 T allele was correlated with lower mRNA expression of the nearby gene *U2AF1* in whole blood, whereas it showed increased mRNA expression of the nearby gene *CBS* in cultured fibroblast cells. These findings implied that the *WDR4* rs6586250 T allele could regulate the expression of multiple surrounding genes. RNA splicing is essential for generating mature messenger RNA (mRNA) and regulating gene expression, thereby affecting cellular processes and cell fates, and is catalyzed by a ribonucleoprotein complex called the spliceosome 47. U2AF1, the subunit of the U2 auxiliary factor (U2AF) heterodimer and an indispensable component of the spliceosome, has the ability to specifically recognize the AG dinucleotide of the 3' splice sites and promote the process of RNA splicing. Furthermore, mutations in *U2AF1* were associated with hematological malignancies and myelodysplasia syndromes 47. According to our analysis, a low expression level of *U2AF1* might affect the activity or efficiency of RNA splicing, leading to the possibility of tumorigenesis. Moreover, we also found a significant association between rs6586250 and the enhanced expression of the neighboring gene* CBS*. Cystathionine β-synthase (CBS) is a mammalian enzyme that transfers sulfur, promoting the conversion of homocysteine to cysteine, and produces hydrogen sulfide (H_2_S), which plays a supportive role in maintaining tumor cell proliferation and survival in cancers with high CBS expression. CBS has been reported to be expressed at a high level in certain cancers, including kidney, colorectal, lung, and breast cancers, but at a low level in liver cancer and glioma. It is predicted that high CBS expression is associated with a poor survival rate for patients with high-CBS-expressing cancers 48. These findings identified *CBS* as an oncogene in several types of tumors, which supports the results of the eQTL analysis. Therefore, it is reasonable to believe that the *WDR4* rs6586250 C > T polymorphism is likely to alter Wilms tumor susceptibility by affecting the expression of nearby genes *U2AF1* and *CBS*. Overall, we speculate that the *WDR4* rs6586250 T allele may increase the risk of Wilms tumor by upregulating *WDR4* gene expression or altering the expression of the surrounding genes *U2AF1* and *CBS*. However, the exact mechanism of how *WDR4* gene SNPs contribute to Wilms tumor susceptibility remains unclear. Further functional experiments are necessary to verify the underlying mechanism.

The strengths of our research include its scientific design, multicenter analysis, and innovativeness. However, we cannot ignore the accompanying disadvantages. First, the low prevalence of Wilms tumor limits the sample size and thus weakens the power of stratified analysis. Most importantly, intensive genetic studies require larger sample sizes and Bonferroni correction analysis to validate the relationship between selected SNPs and tumor risk. Second, the conclusion can only be applied to the Han Chinese, not other countries and ethnicities. Finally, the study focused only on the relationship between *WDR4* gene SNPs and Wilms tumor risk. Therefore, further functional experiments are warranted to explore the unique mechanism by which SNPs in the *WDR4* gene affect Wilms tumor susceptibility.

In brief, this large, five-center epidemiological study demonstrated a significant correlation between functional SNPs in the *WDR4* gene and Wilms tumor susceptibility. The conclusion needs to be further validated in another well-designed scientific study of patients of different ethnicities without confounding factors.

## Supplementary Material

Supplementary tables.

## Figures and Tables

**Table 1:** Relationship of *WDR4* gene polymorphisms with Wilms tumor susceptibility

Genotype	Cases (N=403)	Controls (N=1198)	*P* ^ a^	Crude OR (95% CI)	*P*	Adjusted OR (95% CI) ^b^	*P* ^ b^
rs2156315 C>T (HWE=0.151)							
CC	254 (63.03)	700 (58.43)		1.00		1.00	
CT	129 (32.01)	443 (36.98)		0.80 (0.63-1.02)	0.076	0.80 (0.63-1.02)	0.074
TT	20 (4.96)	55 (4.59)		1.00 (0.59-1.71)	0.994	1.00 (0.58-1.69)	0.985
Additive			0.209	0.88 (0.72-1.07)	0.210	0.88 (0.72-1.07)	0.200
Dominant	149 (36.97)	498 (41.57)	0.104	0.83 (0.65-1.04)	0.104	0.82 (0.65-1.04)	0.100
Recessive	383 (95.04)	1143 (95.41)	0.760	1.09 (0.64-1.83)	0.760	1.08 (0.64-1.82)	0.779
rs2156316 C>G (HWE=0.497)							
CC	187 (46.40)	517 (43.16)		1.00		1.00	
CG	171 (42.43)	548 (45.74)		0.86 (0.68-1.10)	0.227	0.87 (0.68-1.10)	0.240
GG	45 (11.17)	133 (11.10)		0.94 (0.64-1.36)	0.729	0.94 (0.64-1.36)	0.726
Additive			0.407	0.93 (0.78-1.10)	0.407	0.93 (0.79-1.11)	0.414
Dominant	216 (53.60)	681 (56.84)	0.256	0.88 (0.70-1.10)	0.256	0.88 (0.70-1.10)	0.267
Recessive	358 (88.83)	1065 (88.90)	0.972	1.01 (0.70-1.44)	0.972	1.00 (0.70-1.44)	0.983
rs6586250 C>T (HWE=0.296)							
CC	322 (79.90)	945 (78.88)		1.00		1.00	
CT	70 (17.37)	242 (20.20)		0.85 (0.63-1.14)	0.276	0.85 (0.64-1.15)	0.295
TT	11 (2.73)	11 (0.92)		**2.94 (1.26-6.83)**	**0.013**	**2.99 (1.28-6.97)**	**0.011**
Additive			0.759	1.04 (0.81-1.34)	0.757	1.05 (0.82-1.35)	0.717
Dominant	81 (20.10)	253 (21.12)	0.663	0.94 (0.71-1.24)	0.663	0.95 (0.72-1.25)	0.700
Recessive	392 (97.27)	1187 (99.08)	0.007	**3.03 (1.30-7.04)**	**0.010**	**3.08 (1.33-7.17)**	**0.009**
rs15736 G>A (HWE=0.246)							
GG	321 (79.65)	941 (78.55)		1.00		1.00	
GA	74 (18.36)	246 (20.53)		0.88 (0.66-1.18)	0.394	0.89 (0.67-1.19)	0.422
AA	8 (1.99)	11 (0.92)		2.13 (0.85-5.35)	0.106	2.18 (0.87-5.47)	0.098
Additive			0.988	1.00 (0.77-1.29)	0.988	1.01 (0.78-1.30)	0.965
Dominant	82 (20.35)	257 (21.45)	0.639	0.94 (0.71-1.24)	0.639	0.94 (0.71-1.25)	0.678
Recessive	395 (98.01)	1187 (99.08)	0.087	2.19 (0.87-5.48)	0.095	2.23 (0.89-5.59)	0.087
rs2248490 C>G (HWE=0.372)							
CC	187 (46.40)	522 (43.57)		1.00		1.00	
CG	172 (42.68)	548 (45.74)		0.88 (0.69-1.11)	0.279	0.88 (0.69-1.12)	0.301
GG	44 (10.92)	128 (10.68)		0.96 (0.66-1.41)	0.832	0.96 (0.66-1.40)	0.828
Additive			0.496	0.94 (0.79-1.12)	0.496	0.94 (0.80-1.12)	0.509
Dominant	216 (53.60)	676 (56.43)	0.323	0.89 (0.71-1.12)	0.323	0.90 (0.71-1.12)	0.343
Recessive	359 (89.08)	1070 (89.32)	0.896	1.03 (0.71-1.47)	0.895	1.02 (0.71-1.47)	0.913
Combined effect of risk genotypes							
0	182 (45.16)	601 (50.17)		1.00		1.00	
1-5	221 (54.84)	597 (49.83)	0.082	1.22 (0.98-1.53)	0.082	1.22 (0.97-1.53)	0.089

OR, odds ratio; CI, confidence interval; HWE, Hardy-Weinberg equilibrium. ^a^ χ^2^ test for genotype distributions between Wilms tumor patients and controls. ^b^ Adjusted for age and gender. ^c^ Risk genotypes were rs2156315 TT, rs2156316 CC, rs6586250 TT, rs15736 AA and rs2248490 CC.

**Table 2:** Stratification analysis for the association of *WDR4* genotypes with Wilms tumor susceptibility

Variables	rs2156315 (cases/controls)		AOR (95% CI) ^a^	*P* ^a^	rs6586250 (cases/controls)		AOR (95% CI) ^a^	*P* ^a^	Risk genotypes (cases/controls)		AOR (95% CI) ^a^	*P* ^a^
	CC	CT/TT			CC/CT	TT			0	1-5		
Age, month												
≤18	80/273	57/192	1.03 (0.70-1.52)	0.887	134/460	3/5	1.94 (0.46-8.26)	0.370	67/215	70/250	0.89 (0.60-1.30)	0.535
>18	174/427	92/306	**0.73 (0.55-0.98)**	**0.036**	258/727	8/6	**3.89 (1.33-11.37)**	**0.013**	115/386	151/347	**1.45 (1.09-1.92)**	**0.011**
Gender												
Female	112/297	75/224	0.89 (0.63-1.25)	0.490	182/519	5/2	**7.13 (1.37-37.06)**	**0.020**	87/258	100/263	1.13 (0.81-1.58)	0.479
Male	142/403	74/274	0.77 (0.56-1.06)	0.108	210/668	6/9	2.13 (0.75-6.05)	0.157	95/343	121/334	1.29 (0.95-1.76)	0.104
Clinical stage												
I+II	157/700	94/498	0.84 (0.64-1.12)	0.234	244/1187	7/11	**3.11 (1.19-8.15)**	**0.021**	117/601	134/597	1.13 (0.86-1.48)	0.386
III+IV	86/700	49/498	0.79 (0.55-1.14)	0.210	131/1187	4/11	**3.28 (1.02-10.52)**	**0.046**	59/601	76/597	1.34 (0.93-1.92)	0.115

AOR, adjusted odds ratio; CI, confidence interval. ^a^ Adjusted for age and gender, omitting the corresponding factor.

## Abbreviations

m7G: N7-methylguanosine
SNP: single nucleotide polymorphism
MAF: minor allele frequency
LD: linkage disequilibrium
TFBS: transcription factor binding site
ESE: exonic splicing enhancer
ESS: exonic splicing silencer
nsSNP: nonsynonymous coding SNP
HWE: Hardy-Weinberg equilibrium
OR: odds ratio
CI: confidence interval
eQTL: expression quantitative trait locus
GTEx: Genotype-Tissue Expression
GWAS: genome-wide association study
HCC: hepatocellular carcinoma
EMT: epithelial-mesenchymal transition
mRNA: messenger RNA
U2AF: U2 auxiliary factor
CBS: cystathionine β-synthase
