# Supplementary material for: WDR4 gene polymorphisms and Wilms tumor susceptibility in Chinese children: A five-center case-control study
Source: J Cancer. 2023 May 8;14(8):1293–300. doi: 10.7150/jca.83747 (PMC10240673; doi:10.7150/jca.83747)
Supplement: Supplementary file 1 — Supplementary tables. [file jcav14p1293s1.pdf]

**Table S1**. Frequency distribution of selected variables in Wilms tumor patients and cancer-free controls

| Variables | Cases (N=414) | | Controls (N=1199) | | *P* [a] |
|---|---|---|---|---|---|
| | No. | % | No. | % | |
| Age range, month | 1-148.63 | | 0.03-156 | | 0.118 |
| Mean ± SD | 31.14 ± 24.27 | | 32.31 ± 26.15 | | |
| ≤18 | 143 | 34.54 | 466 | 38.87 | |
| >18 | 271 | 65.46 | 733 | 61.13 | |
| Gender | | | | | 0.218 |
| Female | 194 | 46.86 | 520 | 43.37 | |
| Male | 220 | 53.14 | 679 | 56.63 | |
| Clinical stages | | | | | |
| I | 137 | 33.09 | / | / | |
| II | 116 | 28.02 | / | / | |
| III | 94 | 22.71 | / | / | |
| IV | 49 | 11.84 | / | / | |
| NA | 18 | 4.35 | / | / | |

SD, standard deviation; NA, not available.

[a] Two-sided $\chi^2$ test for distributions between Wilms tumor patients and cancer-free controls.

**Table S2**. The potential functions of the selected SNPs from SNPinfo Web Server (https://snpinfo.niehs.nih.gov/snpinfo/snpfunc.html)

| No. | rs | Chromosome | Position | Allele | TFBS | Splicing (ESE or ESS) | miRNA (miRanda) | miRNA (Sanger) | nsSNP |
|---|---|---|---|---|---|---|---|---|---|
| 1 | rs15736 | 21 | 43146927 | G/A | -- | Y | -- | -- | Y |
| 2 | rs2156315 | 21 | 43142750 | C/T | -- | -- | Y | -- | -- |
| 3 | rs2156316 | 21 | 43142894 | C/G | -- | -- | Y | Y | -- |
| 4 | rs2248490 | 21 | 43166813 | C/G | -- | Y | -- | -- | Y |
| 5 | rs6586250 | 21 | 43143298 | C/T | -- | -- | -- | -- | Y |

TFBS, transcription factor binding site; ESE, exonic splicing enhancer; ESS, exonic splicing silencer; nsSNP, non-synonymous coding SNP.
